# In vitro osteogenic induction of human adipose stem cells co-treated with betaine/osteogenesis differentiation medium

**DOI:** 10.22099/mbrc.2021.39354.1578

**Published:** 2021-06

**Authors:** Tayebeh Sadat Tabatabai, Maryam Haji-Ghasem-Kashani, Meysam Nasiri

**Affiliations:** Department of Cellular and Molecular Biology, School of Biology and Institute of Biological Sciences, Damghan University, Damghan, Iran

**Keywords:** Human adipose, derived stem cells, Betaine, Osteogenesis, In vitro

## Abstract

Human adipose-derived stem cells (hADSCs) are widely used in regenerative medicine and affected by many biochemical and biophysical stimuli in vivo. Betaine has been reported to be a type of osteogenic stimulating biochemical factor. This study aimed to investigate the effects of betaine; on osteogenic differentiation of cultured hADSCs in osteogenesis differentiation medium. Mesenchymal stem cells were extracted from women undergoing liposuction after obtaining written consent and cultured in vitro. The cells at passage 4 were confirmed by flow cytometry and differentiated into osteocytes and adipocytes. Experimental groups were the cells cultured in osteogenesis differentiation medium (control), cultured in α-MEM and 10% serum-containing Betaine (BET) ,and cultured in osteogenesis differentiation medium containing 10 mM Betaine (OD+BET). After 14 and 21 days of treatment, osteogenic differentiation and the expression of *RUNX2 *and* OCN* genes were assessed by qualitative and quantitative Alizarin red staining and real-time PCR. There were significant increases in the calcium matrix deposits, alkaline phosphatase activity ,and expression of *RUNX2* and *OCN* genes in the OD+BET group compared to the BET group. At the end of day 14, the calcium matrix formation was significantly decreased the in BET group compared to the control. Treatment of hADSCs with Betaine, and osteogenesis differentiation medium leads to increased alkaline phosphatase activity, matrix calcium deposits and expression of *RUNX2* and *OCN* genes and finally stimulated osteogenesis. This kind of treatment could be used to support bone regeneration in the future of tissue engineering.

## INTRODUCTION

The use of stem cells in tissue engineering is the basis for the emergence of regenerative medicine. Two broad types of stem cells, including embryonic and adult stem cells, have so far been identified [1-3]. The differentiation potentials and therapeutic potentials of embryonic stem cells are very high. The use of these cells in tissue engineering and regenerative medicine is ethically and legally controversial [4-6]. Researchers have focused more on the use of adult stem cells. Adipose tissue is a rich source of adult stem cells [1]. 

One of the ideal characteristics of stem cells extracted from adipose tissue is that they retain their differentiation potential in long-term culture or successive passages. Aging has more negligible effect on adipose tissue-derived stem cells (ADSCs) than on bone marrow-derived stem cells (BMSCs) [4, 7, 8]. There is also no significant difference in cell adhesion, aging, differentiation capacity, and gene transfer between ADSCs and BMSCs [9]. Tissue regeneration using human adipose-derived stem cells (hADSCs) also has significant potential in the treatment of bone and joint disorders [10]. Clinical transplantation of ADSCs is currently the most promising field for bone regeneration in the laboratory [3]. 

From 2013 onwards, roughly 3000 journals have been investigated the effectiveness and safety of ADSCs in regenerative medicine [11]. The findings suggested the in vitro osteogenic ability of ADSCs post-induction and their potential therapeutic applications in bone defects [12]. In 2003, Lee et al. observed the first evidence of bone formation in living organisms after differentiation of ADSCs into osteoblasts in the laboratory [4]. Because of these benefits, hADSCs have been used in this study. 

Growth factors, biomolecules, or small inorganic molecules are used as chemical compounds to stimulate bone tissue regeneration. Betaine is a biochemical stimulant (a dietary supplement) that stimulates the proliferation and differentiation of osteoblasts. The main physiological role of betaine is as an osmolyte and donor of a methyl group. As an osmolyte, betaine by betaine/GABA transporter-1 (BGT1) protected from the cells, proteins, and enzymes against environmental stress (dehydration, high salinity, or high temperature) [13-17]. It also maintains fluid balance. As a methyl donor, it converts homocysteine to methionine by the enzyme betaine-homocysteine, and also produces N, N-dimethylglycine [13, 14, 18, 19]. 

Betaine is an effective and safe treatment for patients with genetic homocystinuria, alcoholic and non-alcoholic fatty liver diseases [20-23]. It has been reported that there is inverse association between betaine dietary intakes and lung, colon ,and breast cancers in humans [24]. In humans, betaine has been reported to improve athletic performance. Recent research has shown that betaine supplementation can increase muscle strength and function. Betaine may also increase muscle mass in trained men, but its mechanisms have not been identified [25]. Evidence suggests that betaine, as a trimethylglycine derivative, regulates several vital biological processes such as oxidative stress, inflammatory, osteoblast differentiation, and cellular apoptosis [26-28].

Therefore, considering the role of betaine in osteoblast differentiation, this study aimed to investigate the effects of betaine on osteogenic differentiation of human adipose-derived stem cells. 

## MATERIALS AND METHODS

**Isolation and culture of human ADSCs: **To isolate hADSCs, adipose tissue samples were collected from women (mean age 40±5) undergoing liposuction. Informed consent was obtained from the participants and approval of the local Ethics Committee at Velayat Hospital (Damghan, Iran). The study was carried out following the guidelines of the Medical Ethics Committee, Ministry of Health of Iran. Firstly, the sample was cut into very small pieces and then 0.2% collagenase (Gibco, 17100-017) was added, and incubated at 37°C for 2 h. To stop digestion, DMEM (Invitrogen, USA) containing 10% FBS was added to the suspension and centrifuged at 1200 rpm for 5 min at 37°C. Finally, the isolated cells were transferred and cultured in a 25 cm^2^ flask, and incubated at 37°C in 5% CO_2_ for 72 h. hADSCs adhered to the bottom of the flask, while floating blood cells washed away by replacing the medium with a fresh one. Cells were subcultured and used for experiments at passage 4 [4].

**Flow cytometry analysis: **Flow cytometric analyses were performed on a FACSCalibur (BD biosciences, USA). Passage 4 of hADSCs was trypsinized, and then centrifuged at 1200 rpm for 3 min at RT and resuspended in FACS buffer (PBS, 2% FBS) and incubated on ice for 10 min. Then fluorescence antibodies against CD73-PE (BD Biosciences, cat. No. 562817), CD105-PE (Exbio, cat. No. 1P-298-T025), CD166-FITC (Exbio, cat. No. 1F-652-100T), CD45-FITC (BD Biosciences, cat. No. 560976) and CD34-PE (Exbio, cat. No. 1P-664-T025) were added and incubated with rotation at 4°C for 30 min. After removing of non-conjugated antibodies by three washes, the cells were resuspended in PBS. The expression of each surface marker was assessed [29].

**Induction** **of osteoblast differentiation: **The isolated hADSCs at passage four (P4) were cultured in 12- well plates, and medium were replaced with Osteogenesis Differentiation Medium (StemPro® Osteogenesis Differentiation Kit, A10072-01, Invitrogen); about 21 days later, the cells were stained with Alizarin red method. Briefly, cells were fixed with 4% formaldehyde for 10 min at 4°C and then incubated with Alizarin Red for 2 min. Then cells were washed with PBS and observed by inverted microscope (E600-Eclipse Nikon) equipped with a digital camera (DXM 1200 Camera Nikon Digital) [30].

**Induction of adipocyte** **differentiation:**The isolated hADSCs at P4 were subcultured in 12- well plates containing Adipogenesis Differentiation Medium (StemPro®Adipogenesis Differentiation Kit, A10070-01, Invitrogen). After 21 days, the cells were fixed and stained with Oil Red-O. Briefly, cells were washed with PBS two times and then fixed with 4% formaldehyde for 1 h at 4°C. Then the cells were washed with 70% ethanol for 10-15 min, and then stained with Oil Red. Finally, cells were washed with 70% ethanol about three times and microscopic observation was done to check the results [7].

**Experimental groups: **The studied groups were: cultured P4- cells in Osteogenesis Differentiation Medium (ODM), cultured P4-cells in α-MEM (α-Minimal Essential Medium, Invitrogen USA) containing 10% FBS and 10 mM of betaine( (20, 26, 31) (Betain Cat No: K1311, a product of Kawsar Biotech Company in Iran) and cultured P4- cells in ODM and 10 mM of betaine (OD+BET) 

**Alizarin Red staining and calcium deposit quantification: **To identify the mineralized matrix of differentiated cells, Alizarin Red (069K1639, Sigma-Aldrich) staining was used on day 21. After removing the medium, the samples were washed with cold PBS and fixed in cold 4% paraformaldehyde (Merck, Germany) for 20 min at 4˚C. Fixative was removed ,and cells were washed twice with PBS. The fixed cells were stained with 400µl Alizarin Red at pH 7.2 for 5-10 min. Finally, the cells were washed again with PBS for three times and observed by inverted microscope [32]. For calcium quantification, 300 µl of 10% acetic acid was added to the cells stained with Alizarin Red and incubated for 30 min at RT with agitation. Cells were scraped off, transferred to microtubes, vortexed ,and incubated. Samples were centrifuged for 30 min at 2000 rpm ,and then 200 µl of the supernatant was transferred to another microtube, 22.5 µl of 10% ammonium hydroxide was added to neutralize the acid and mixed. Finally, the absorbance was measured at 405 nm by the BioTek device [33].

**Alkaline Phosphatase activity: **To measure alkaline phosphatase (ALP) activity, the total protein of the cells at days 7, 14 post-induction, were lysed with 30 μl of Triton X-100 lysis buffer. The lysate was centrifuged at 2000 rpm at 4°C for 10 min and the ALP activity of supernatant was measured using para-nitrophenyl phosphate (pNPP) as a phosphatase substrate (ALP Kit, Iran) at 405 nm by a microplate reader (BioTek Instruments, USA). Finally, the enzyme activity level (IU) was normalized against the total protein [34].

**Calcium content assay: **To evaluate the amount of calcium deposits of induced cells on day 21, the samples were washed with PBS and homogenized in 500 μl 0.6 N HCl (Merck, Germany), which were followed by shaking for 4 h at 4°C. Then, a reagent (Calcium Content Kit, Iran) was added to calcium solutions, and the optical density of samples was measured at 575 nm, by a microplate reader (BioTek Instruments, USA). In the end, the value of calcium contents was extracted from the standard curve of OD against a serial dilution of calcium concentrations [35].

**Gene expression analyses using RT-qPCR: **Total RNA was extracted using RNX-plus reagent (Sinaclon, Iran) according to the manufacturer’s protocol. The cDNA was synthesized using the PrimeScript™ 1st strand cDNA Synthesis Kit (Takara, Japan). Real-time PCR was done by Rotor-Gene 6000 PCR system, using RealQ Plus Master Mix Green (Amplicon, Denmark). The final volume of the reaction solution was 10μl and the program set as, denaturation at 95°C for 15min followed by 50 cycles at 95°C for 15s and annealing/extension for 45s at 60°C [36, 37]. Primer sequences were designed by AlleleID software version 7.5 (Premierbiosoft, USA) as shown in Table 1. The relative expression of target genes was calculated through the 2^-ddCt^ formula, and the RPL13a gene was selected as an endogenous control.

**Table 1:** Gene expression primer sequences and amplicons

**Gene**	**Accession number**	**Primer sequence (5** ^'^ **→3** ^'^ **)**	**Amplicon size (bp)**
RUNX2	NM_001015051	AATGCCTCTGCTGTTATGAA CTTCTGTCTGTGCCTTCTG	192
BGLAP	NM_199173	ACCGAGACACCATGAGAG CCAGCCATTGATACAGGTAG	183
RPL13a	NM_012423	GATAAGAAACCCTGCGACAAA AGAAATTGCCAGAAATGTTGATG	193

**Statistical Analysis: **All data is shown as mean ± standard error. Data analysis was performed using SPSS software version 16. The differences between the experimental groups were analyzed using one-way ANOVA test followed by Tukey post-hoc test and P<0.05 was considered as a significant level.

## RESULTS

**Figure 1:**
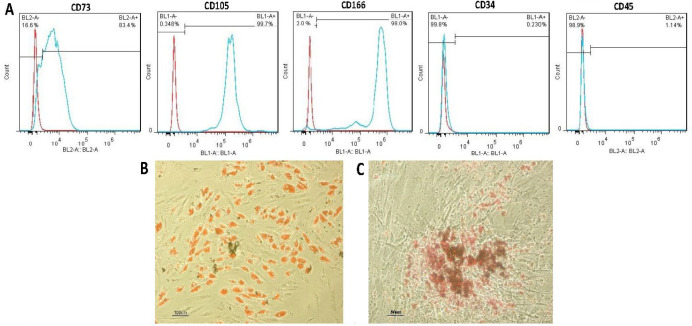
Identity of isolated hADSCs. (A) The cells positively expressed CD73, CD166, CD105 markers, while being negative for CD34 and CD45. (B) Oil Red staining of adipogenic-induced cells shows intracellular lipid droplets. (C) Representative images of Alizarin Red staining at 21 days of osteogenic induction, mineralized calcium deposits are in red

Purified MSCs from human adipose tissue could be characterized by the expression of surface markers. As shown in figure 1, about 83.4% of the cells responded positively to the CD73 marker, and about 98% of the cells responded positively to the CD166 marker. This rate reached about 99.7% for the CD105 marker. Isolated cells were negative in terms of CD34 and CD45 markers (Fig. 1A). Also, hADSCs demonstrated a strong capacity for differentiation into adipogenic and osteogenic lineages. Adipogenic differentiation was confirmed by the appearance of small lipid vesicles formed after treatment and stained with Oil Red (Fig. 1B). Osteogenic differentiation was confirmed by the production of calcium phosphate and mineralized extracellular matrix in induced cells, which were stained with Alizarin Red (Fig. 1C). 

**Figure 2:**
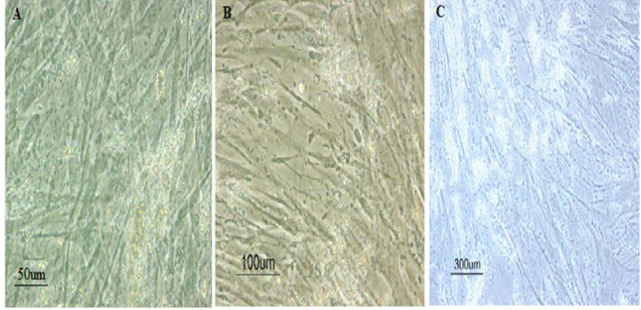
Morphology of the studied groups. A: control, B: BET, C: OD+BET

**Figure 3:**
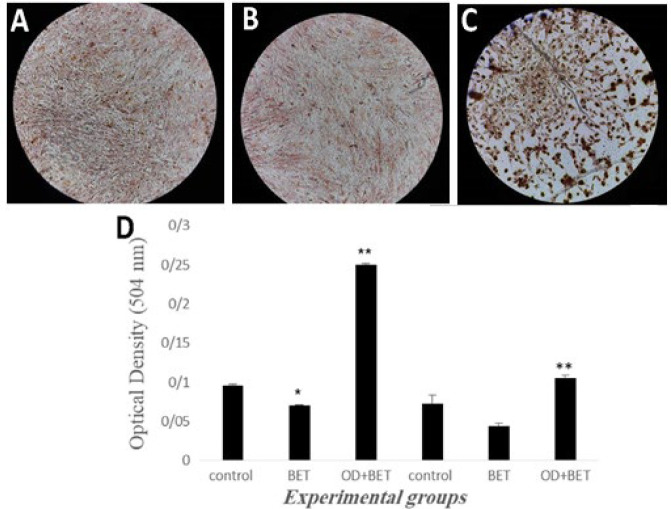
Extracellular matrix calcium deposits were visualized with Alizarin Red staining. A: control, B: BET and C: OD+BET groups. Magnification 100 .×D: Diagram of the amount of calcium phosphate through quantitative staining of Alizarin Red. Control (cultured cells in Osteogenesis Differentiation Medium); BET (cultured cells in α-MEM containing 10% FBS and Betaine); OD+BET (cultured cells in Osteogenesis Differentiation Medium containing Betaine). *Significant difference versus control, **Significant difference versus BET group

The cells appeared triangular or polygonal shaped after treatment with OD and betaine, the formation of calcium deposits and mineralized matrix was higher in the OD + BET group (Fig. 2C) compared to control and BET groups (Fig. 2A & B). Alizarin Red (red indicative of calcium deposition) staining showed more intense staining for mineral in the OD + BET group (Fig. 3C) compared to those of other groups, while there was no difference in the formation of calcium deposits between the control and BET groups (Fig. 3A & B).

The quantitative analysis of Alizarin Red staining after 14 and 21 days of induction was shown in fig. 3D. On day 14, BET group (0.05) showed a significant decrease of calcium deposits, compared to the control group (0.095±0.002) (P<0.05). But it was significantly higher in OD+BET group (0.250±0.001) compared to the BET group (P<0.05). On day 21, no significant difference of calcium deposits could be detected among the control (0.070±0.010) and BET groups (0.044±0.003). In contrast, OD+BET group (0.105±0.003) showed a significant increase of calcium deposits, compared to the BET group (P<0.05). 

**Figure 4:**
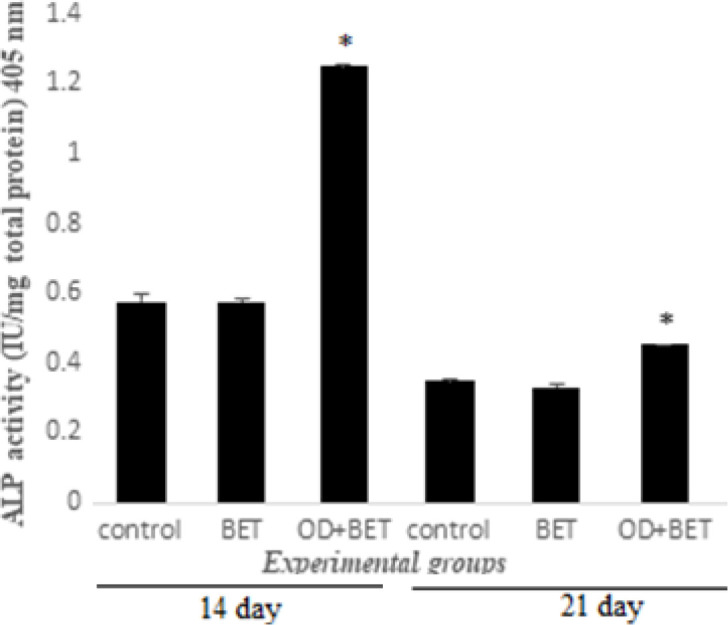
ALP expression rate of the experimental groups on day 14 and 21. * significant increase versus BET group

The ALP activity of the experimental groups was measured on days 14 and 21 post-induction (Fig. 4). After 14 days, the ALP activity of the BET group (0.573±0.008) showed no difference from that of the control (0.575±0.022). Whereas ALP activity of the OD+BET group (1.248±0.007) was significantly increased, compared to the BET group. After 21 days, no significant difference in ALP activity could be detected among the control (0.349±0.002) and BET (0.329±0.010) groups. While OD+BET group (0.451±0.001) showed a significant increase in ALP activity, compared to BET groups. 

**Figure 5:**
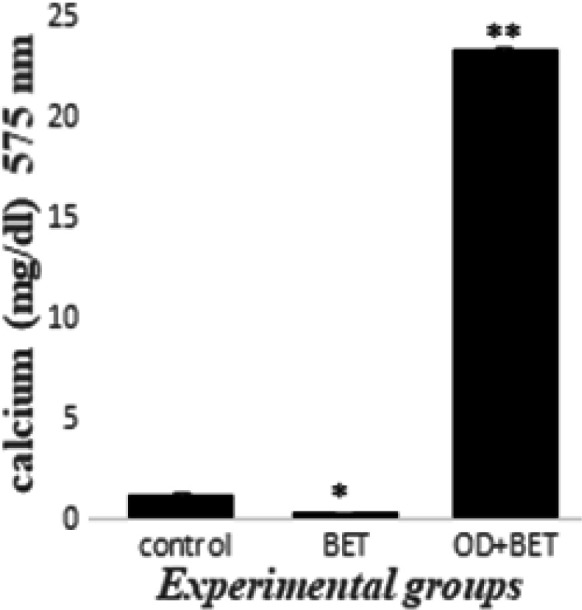
The amount of calcium deposits in experimental groups on day 21.*Significant decrease versus control group, **Significant increase versus BET group

The amount of calcium deposition of experimental groups was evaluated on day 21 (Fig. 5). There was a significant decrease in calcium deposition in the BET group (0.350) compared to that of the control group (1.187±0.116). There was a significant increase in calcium deposition in OD+BET (23.330±0.120) group as compared to that of the BET group (P<0.05). 

**Figure 6:**
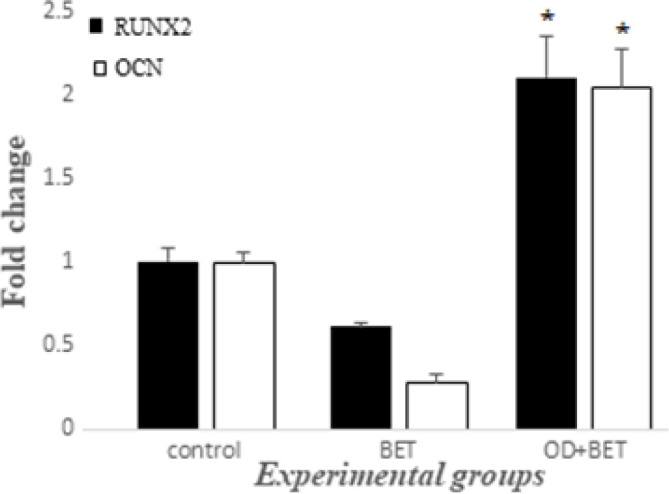
The expression of *RUNX2* and *OCN* genes in hADSCs, 21 days post-induction in experimental groups. *Significant increase versus BET group

According to Figure 6, after 21 days no significant difference in the *RUNX2 *mRNA expression level, could be detected among the control (1.003±0.0788) and BET (0.613±0.0233) groups. While there was a significant increase in the mRNA level of the *RUNX2 *gene in the OD+BET group (2.10±0.251) compared to the BET group. Also, after 21 days no significant difference in the *OCN *mRNA expression level, could be detected among the control (1±0.060) and BET (0.276±0.050) groups. While there was a significant increase in the mRNA level of the *OCN* in the OD+BET group (2.04±30.230) compared to the BET group.

## DISCUSSION

The importance physiological function of betaine as an osmoprotectant and methyl group donor indicating its deficiency associated with adverse consequences. Betaine is added to animal diets to improve meat quality (fat loss and muscle gain) [38]. Betaine has been reported as a promising therapeutic agent against sarcopenia and osteoporosis (a major cause of bone weakness and mortality) [20]. Betaine has been previously reported to induce stimulatory effects on human osteoblasts and myoblasts by synergistic activation of IGF-1 production. It leads to the expression of genes and ultimately the production of matrix proteins [20, 31, 39, 40]. It has also been shown that betaine supplements may be important in bone and muscle disorders in the elderly by acting on bone and muscle cells through common pathways [41]. Therefore, the aim of this study was to investigate the effects of betaine on the osteogenic differentiation of hADSCs. 

Evidence suggests that betaine depolarizes osteoblast plasma membranes through the VGCC type L channel, thus exerts osteogenic effects. Betaine has been reported to affect osteoblasts by increasing intracellular Ca^2 +^ levels, ECM production, insulin-like growth factor (IGF-2), and a series of events leading to osteogenesis in vitro. IGF-I has been reported to stimulate the *RUNX2* gene expression during osteogenesis. The *RUNX2* gene is the major transcription factor of the osteogenesis process. 

RUNX2 can stimulate other osteoblast-specific transcription factors such as Osterix (OSX) followed by the activation of *BSP* and *OPN* (two non-collagen proteins). These proteins are multifunctional, having roles in matrix mineralization, bone formation, and regeneration [20, 42-47]. 

The cells exhibited a polygonal or triangular morphology. The calcium deposits stained by Alizarin Red were observed in OD + BET with the high amount and BET groups (two main characteristics during the osteogenic differentiation). Thus, it can be argued that the betaine/ODM enhanced osteogenic activity of hADSCs. Similar to this study, Villa observed changes in the morphology of human osteoblast-like cells (hOBs) post induced by betaine and osteogenic medium. He also suggested that betaine increases the osteogenic differentiation of hOBs [20]. 

hADSCs are detected by the expression of surface antigens such as CD73, CD166 and CD105, and by the lack of hematopoietic antigens such as CD34, CD45 [48-50]. The efficacy of hADSCs studied in this study was confirmed by examining these markers and the results were by Zhu reports [51]. The results showed that hADSCs differentiate into osteoblasts and adipocytes, similar to Tapp's and Locke's findings [4, 52]. 

Cultured hADSCs in ODM for 14 to 28 days, produced the mineralized calcium phosphate in the extracellular matrix stained by Alizarin Red [3]. Analysis of the quantification of Alizarin Red staining after 14 and 21days, showed the increased osteogenic potential of hADSCs post induced by betaine/ODM. These results are in line with those of Villa study. He reported that the co-treatment of betaine ,and the osteogenic medium through the RUNX2/OSX axis leads to the activation of bone formation markers such as type-1 collagen, *BSP* and *OPN.* Expression and activation of these proteins lead to matrix mineralization, bone formation and regeneration. Treatment with betaine alone did not induce osteogenesis of hADSCs. 

The present study showed that after the end of days 14 and 21, co-treatment of these cells with ODM and betaine increased the ALP activity. These findings are similar to Villa's results. He observed a significant increase in ALP activity and osteocalcin gene expression after treatment of hOBS with betaine (10 mM) and ODM. Treatment of hADSCs alone with betaine exhibited a significant reduction of alkaline phosphatase activity at 14 and 21 days. In fact, it can be claimed that betaine alone does not affect the osteogenic differentiation potential of hADSCs. After 21 days of cell incubation and betaine treatment, the stimulatory effects of betaine /ODM on osteogenesis, similar to the Villa study were determined. He reported that betaine (10 mM) increased intracellular calcium by the flow of calcium ions through L-type VGCC channels. Also, treatment with betaine alone had inhibitory effects on osteogenesis of hADSC. 

Betaine has been reported to be effective in the proliferation and differentiation of osteoblasts in the laboratory by stimulating DNA synthesis and the expression of osteogenesis-related genes during differentiation and matrix mineralization [20]. At the end of days 14 and 21, we found the stimulatory effect of ODM betaine on the expression of *RUNX2* and *OCN* genes. Data analysis also indicates that betaine alone may have no affect on the expression of *RUNX2 *and *OCN* genes.
